# Extrapleural solitary fibrous tumor—a revision of three cases

**DOI:** 10.1093/jscr/rjad151

**Published:** 2023-03-28

**Authors:** Filipa Policarpo, Ana Alves Rafael, José Nuno Teixeira, Martinha Chorão

**Affiliations:** General Surgery,Centro Hospitalar Lisboa Ocidental, Lisboa, Portugal; General Surgery,Centro Hospitalar Lisboa Ocidental, Lisboa, Portugal; General Surgery,Centro Hospitalar Lisboa Ocidental, Lisboa, Portugal; Pathology, Centro Hospitalar Lisboa Ocidental, Lisboa, Portugal

## Abstract

Extrapleural solitary fibrous tumors are a rare type of spindle cell neoplasm, which can occur in many locations with different histologic and immunohistochemical findings, making the diagnosis challenging. They are usually indolent and their treatment is based on complete surgical resection. There are still some issues to be clarified regarding systemic therapy (specifically when aggressive behavior exists) and long-term follow-up. We present a series of clinical cases in the same Department and review this thematic area.

## INTRODUCTION

Extrapleural solitary fibrous tumors (SFT) are a rare type of spindle cell neoplasm [1] more commonly observed between the fifth and seventh decades, with no gender predominance [1, 2]. Despite its initial description as a pleural lesion [1, 3], 70% of SFT occur in an extrapleural location [1]. As SFT can appear in any anatomic site, its clinical presentation depends on its location [1, 2]. With no pathognomonic imaging findings [2], definitive diagnosis is based on cellular morphology and immunohistochemical characteristics [1]. Although extrapleural SFT are classically characterized by spindle hypocellular areas intercalated with hypercellular areas with collagen bundles and prominent vascularization [1, 3, 4], SFT may present as a wide range of histologic findings [1, 3].

Due to this histologic variability, its rarity and evidence based on retrospective studies and case reports, SFT natural history is not completely known [1, 3, 4].

Therefore, the authors propose a review of this thematic area, through a presented case series.

## CASE SERIES

### Case 1

**Figure 1:**
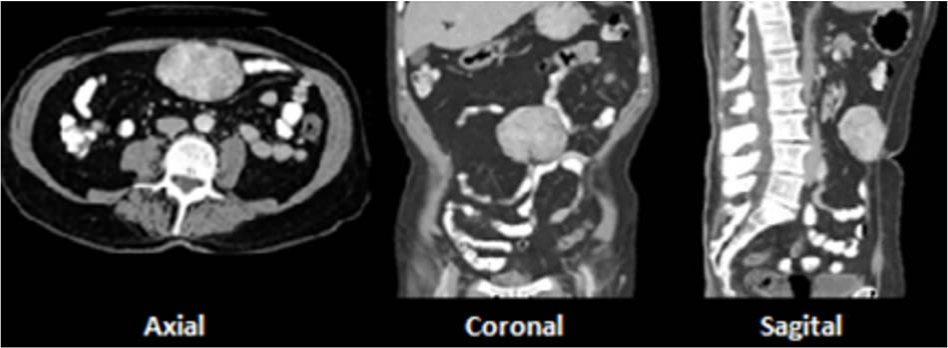
A 9-cm solid preperitoneal lesion at umbilical level.

A 73-year-old male with prior history of irritable bowel syndrome presented a 2-week-long abdominal pain, located on the right lower quadrant associated with anorexia and diarrhea, without blood loss or any other symptoms. The CT-scan showed a 9-cm solid preperitoneal lesion, with enhancement following intravenous contrast administration (Fig. 1).

Patient was submitted to *en bloc* excision. The histologic analysis revealed a completely excised SFT of 9 × 7.5 × 4 cm, with no malignant signs.

After multidisciplinary discussion, the patient was kept under surveillance. No signs of recurrence were reported during the 4 years of follow-up.

### Case 2

**Figure 2:**
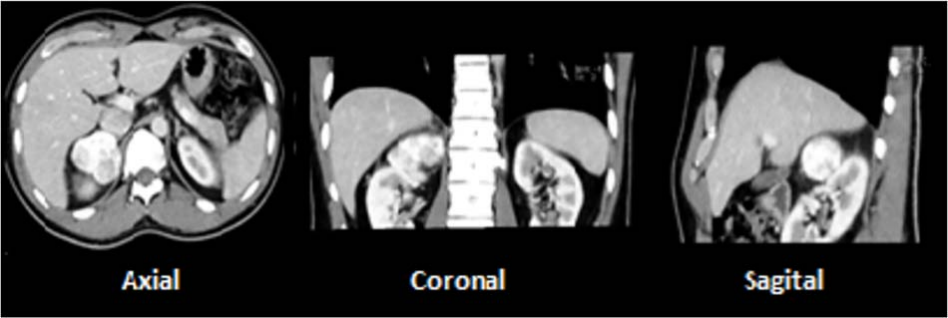
A 5.4-cm lesion in right adrenal gland.

A 28-year-old male with prior history of symptomatic renal lithiasis. During clinical investigation of a persistent right lumbar pain with no other symptoms, a 54 × 43 × 37-mm non-functional adrenal incidentaloma was detected, with an absolute washout of 64% and relative washout of 56%, suggestive of adrenal adenoma (Fig. 2).

After pain control with oral therapy, patient was submitted to an elective laparoscopic right adrenalectomy, without complications. The histologic analysis revealed an SFT totally excised, adherent to a normal adrenal gland.

Currently, patient is kept under surveillance, with no signs of recurrence during the past 3 years of follow-up.

### Case 3

A 59-year-old male presented a multinodular goiter, without hypo- or hyperthyroidism or compressive symptoms. The ultrasound showed a solid, hypoechogenic and well-defined 8 × 6 × 4-cm dominant node. The preoperative cytologic analysis was non-diagnostic. The patient was then submitted to total thyroidectomy, without complications. The histologic and immunohistochemical findings diagnosed an 8 cm completely excised SFT, with no signs of malignancy.

Still under surveillance after multidisciplinary discussion, the patient has presented no signs of recurrence during the 2 years of follow-up.

We present a table that summarizes the case series description (Table 1).

**Table 1:** Summary of the case series description (IHQ – immunohistochemical analysis; M – male)

Case	Gender	Age	Location	IHQ		Treatment	Follow-up
1	M	73	Preperitoneal	Bcl2 + CD34+ CD99+ STAT6+	S100 – CD117 –	Excision	4 years follow-up: no recurrence
2	M	28	Adrenal gland	Vimentin + Bcl2 + focal CD34 + focal STAT6+	Actin – S100 –	Excision	3 years follow-up: no recurrence
3	M	59	Thyroid	Vimentin + Bcl2 + CD34+ Actin + focal STAT6+	S100 – EMA –	Excision	2 years follow-up: no recurrence

## DISCUSSION

Extrapleural SFT are more commonly observed between the fifth and seventh decades, with no gender predominance [1, 2] or known risk factors [1].

SFT can appear in any anatomic site and its presentation depends on its location [1, 2]:

- Abdomen: most frequent symptoms include a palpable mass, pain and abdominal distension. Due to the free space of the abdominal cavity and the capacity of distension of its wall, symptomatic patients usually present large tumors, with compressive effect against the surrounding structures [2].
- Meninges: generally intracranial tumors, which cause compression of surrounding structures or high intracranial pressure [2].
- Head and neck: usually small lesions easily recognized through a painless mass. When symptomatic, they can present as proptosis or reduced visual acuity [2].
- Soft tissues of the extremities: generally present as a painless mass.

Despite there being no pathognomonic imaging findings, typically SFT present as soft tissue density, homogeneous and well-defined mass, occasionally with cystic areas, calcifications or bleeding, causing displacement, but no invasion of adjacent structures [2].

Definitive diagnosis is based on cellular morphology and immunohistochemical characteristics [1].

**Figure 3:**
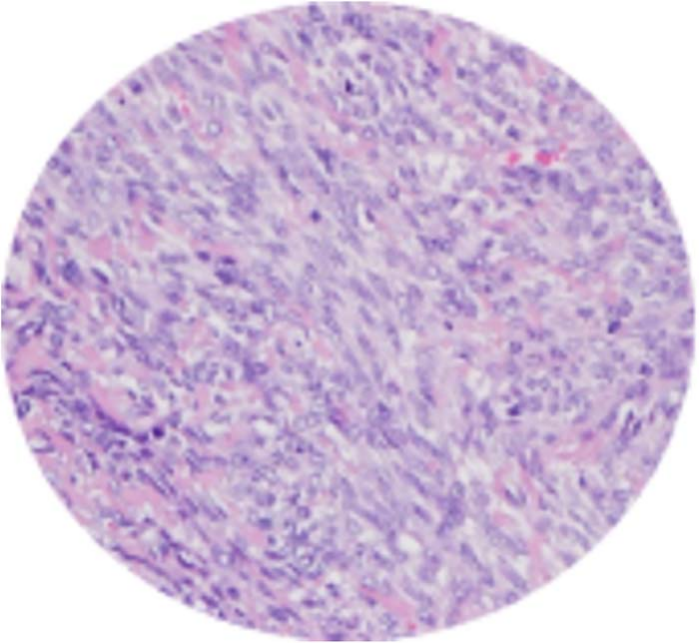
Spindle cell neoplasm with collagen bundles and capillary vascularization.

Extrapleural SFT are classically characterized by spindle hypocellular areas intercalated with hypercellular areas with collagen bundles and prominent vascularization [1, 3, 4] (Fig. 3). However, SFT may present as a wide range of histologic findings, from spindle hypocellular areas to hypercellular variants with round shape and low-collagen cells, making the diagnosis challenging [1, 3].

**Figure 4:**
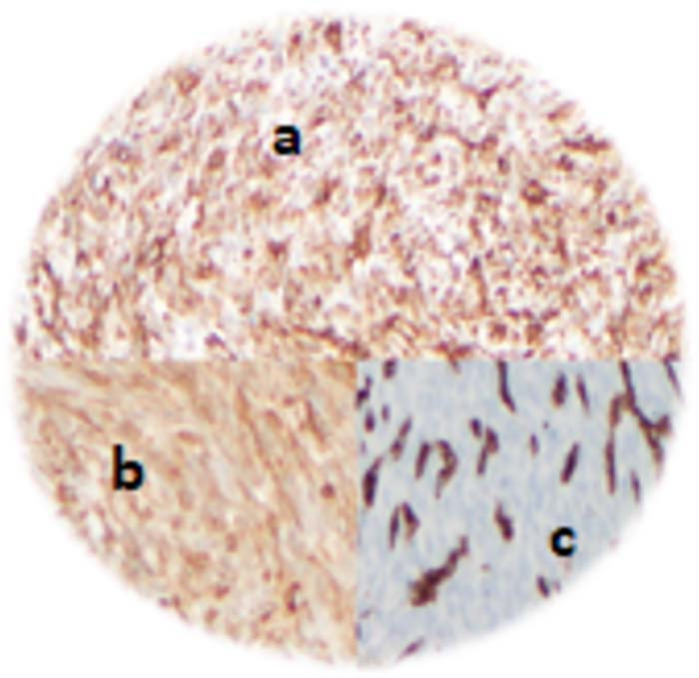
Immunohistochemical analysis – vimentin+ **(a)**, Bcl-2+ **(b)** and CD 34+ **(c).**

There are some biomarkers that can help diagnosis, although they are not specific for SFT. Usually SFT stain positive for vimentin, Bcl2, CD34, CD99 and STAT6, but not for actin, desmin or S100 [1] (Fig. 4).

Despite indolent growth of most extrapleural SFT [1–3], benign and malignant distinction is not clearly defined, due to histologic variability and mismatch between histologic findings and tumoral aggressiveness [1].

However, some parameters are associated to more aggressive behavior, metastasis and recurrence, namely:

- size >10 cm
- >4 mitoses per 10 high-power fields
- tumoral necrosis
- positive margins [4].

The treatment of localized extrapleural SFT is based on complete resection [1, 3, 4]. Adjuvant therapy is not recommended, since there is no evidence of benefit in this situation [1].

Recommendation of systemic therapy in unresectable or metastatic disease is not clearly defined, despite some successful reported cases with antiangiogenic therapy [2]. Hence, the importance of multidisciplinary decisions.

The estimated rate of metastatic disease is 6–23%, more frequently to lung, bone and liver [1].

Therefore, long-term follow-up is advisable [1, 3], due to the indolent natural history of SFT, even in recurrence. However, the surveillance strategy is not currently well-defined in the literature.

In the reported cases, a semestral clinical evaluation and annual CT-scan in abdominal SFT was performed. The patient with SFT in the thyroid gland underwent annual ultrasound and scintigraphy and MRI 1 year after the surgery.

In conclusion, extrapleural SFT remains a rare and challenging disease, raising questions regarding treatment and long-term follow-up.

Consequently, an international compilation of reported cases seems necessary, aiming at more detailed study and the further creation of international recommendations.

## Data Availability

All data are presented in the main manuscript or additional supporting files.
